# Hydrogen Sulfide Ameliorates SARS-CoV-2-Associated Lung Endothelial Barrier Disruption

**DOI:** 10.3390/biomedicines11071790

**Published:** 2023-06-22

**Authors:** Olivier Escaffre, Peter Szaniszlo, Gabor Törő, Caitlyn L. Vilas, Brenna J. Servantes, Ernesto Lopez, Terry L. Juelich, Corri B. Levine, Susan L. F. McLellan, Jessica C. Cardenas, Alexander N. Freiberg, Katalin Módis

**Affiliations:** 1 Department of Pathology, University of Texas Medical Branch, Galveston, TX 77555, USA; 2 Institute for Human Infections & Immunity, Sealy & Smith Foundation, University of Texas Medical Branch, Galveston, TX 77555, USA; 3 Department of Surgery, University of Texas Medical Branch, Galveston, TX 77555, USA; 4 John Sealy School of Medicine, University of Texas Medical Branch, Galveston, TX 77555, USA; 5 Department of Anesthesiology, University of Texas Medical Branch, Galveston, TX 77555, USA; 6 Department of Internal Medicine, Division of Infectious Diseases, University of Texas Medical Branch, Galveston, TX 77555, USA; 7 The Center for Translational Injury Research, Department of Surgery, UTHealth McGovern Medical School, Houston, TX 77030, USA; 8 The Center for Biodefense and Emerging Infectious Diseases, University of Texas Medical Branch, Galveston, TX 77555, USA

**Keywords:** endothelial barrier, COVID-19, SARS-CoV-2, hydrogen sulfide, cytokine, TNF-α

## Abstract

Recent studies have confirmed that lung microvascular endothelial injury plays a critical role in the pathophysiology of COVID-19. Our group and others have demonstrated the beneficial effects of H_2_S in several pathological processes and provided a rationale for considering the therapeutic implications of H_2_S in COVID-19 therapy. Here, we evaluated the effect of the slow-releasing H_2_S donor, GYY4137, on the barrier function of a lung endothelial cell monolayer in vitro, after challenging the cells with plasma samples from COVID-19 patients or inactivated SARS-CoV-2 virus. We also assessed how the cytokine/chemokine profile of patients’ plasma, endothelial barrier permeability, and disease severity correlated with each other. Alterations in barrier permeability after treatments with patient plasma, inactivated virus, and GYY4137 were monitored and assessed by electrical impedance measurements in real time. We present evidence that GYY4137 treatment reduced endothelial barrier permeability after plasma challenge and completely reversed the endothelial barrier disruption caused by inactivated SARS-CoV-2 virus. We also showed that disease severity correlated with the cytokine/chemokine profile of the plasma but not with barrier permeability changes in our assay. Overall, these data demonstrate that treatment with H_2_S-releasing compounds has the potential to ameliorate SARS-CoV-2-associated lung endothelial barrier disruption.

## 1. Introduction

As of May 2023, just after the third anniversary of its officially declared outbreak, Coronavirus Disease 2019 (COVID-19) has caused more than 6.9 million deaths worldwide. The causative agent of this disease, Severe Acute Respiratory Syndrome Coronavirus-2 (SARS-CoV-2), has infected over 760 million people globally, and this number is still rising rapidly [1]. Despite substantial advances in prevention and treatment strategies, this pandemic still poses global health and economic challenges due to the constantly emerging novel variants of the virus and the long-term consequences of the infection, collectively termed post-COVID conditions or long COVID [2,3,4]. COVID-19 was initially viewed as a respiratory disease, but growing evidence supports the critical role of endothelial dysfunction not only in the pulmonary vasculature but in other organs, both in acute cases and during long COVID [5,6,7,8,9].

Severe manifestations of COVID-19 are characterized by progressive respiratory failure resulting from diffuse alveolar damage with inflammatory infiltrates and alveolar edema, intra-alveolar fibrin deposition and hemorrhage, endothelialitis, as well as pulmonary and systemic coagulopathy that form obstructive microthrombi in the lung and other organs [6,10,11]. Additional pathological findings in the vasculature of COVID-19 lungs include the disruption of intercellular junctions, basal membrane contact loss, neutrophilic capillaritis/endothelialitis, pulmonary thromboembolism, pulmonary infarctions, and venous thrombosis [12,13]. The contribution of different factors to COVID-19-related endotheliopathy is still under debate. A growing body of evidence points to high levels of pro-inflammatory cytokines and chemokines produced in the lung tissue by infected alveolar epithelial cells and alveolar macrophages [14,15,16], platelet activation [17,18], as well as to direct contact of endothelial cells (ECs) with the SARS-CoV-2 spike protein [19,20,21,22]. At the same time, recent data indicate that direct viral infection of ECs is less likely to play a major role in these processes [12,22,23,24,25].

In the lung, alveolar epithelial cells are surrounded by an extracellular matrix and adjacent pulmonary microvascular ECs, forming the alveolar–capillary endothelial barrier through inter-endothelial junctions [26,27]. The permeability properties of this barrier are tightly regulated through interactions between ECs, surrounding tissue, and biologically active molecules in the blood [26,28]. Lung microvascular endothelial injury has been linked to the most severe complications of COVID-19, acute respiratory distress syndrome (ARDS), multiorgan failure, and death [8,29]. Recent studies have demonstrated that lung endothelial barrier damage and dysfunction—characterized by increased vascular permeability and loss of barrier integrity leading to the leakage of fluid and plasma proteins into the surrounding tissue—play a critical role in the pathophysiology of the disease and contribute to the development of ARDS [8,16,17]. In the current clinical practice, no specific therapeutic strategies aim to restore the endothelial barrier in COVID-19 patients.

Hydrogen sulfide (H_2_S) is a gaseous signaling molecule produced in various mammalian cell types, including ECs [30,31]. Among a wide array of physiological functions, it plays a fundamental role in vascular homeostasis, modulates inflammatory responses, and reduces vascular leakage [30,32,33,34,35]. Several studies have demonstrated that H_2_S improves endothelial barrier function in a variety of experimental conditions. H_2_S inhalation attenuated pathologically enhanced blood–brain barrier permeability in animal models of cardiac arrest and resuscitation [36,37]. H_2_S treatment prevented lipopolysaccharide (LPS)-induced hyperpermeability in EC cultures [38] and protected against LPS inhalation-induced acute lung injury by reducing neutrophil transmigration and inhibiting pro-inflammatory signaling in animal models [39,40]. The H_2_S donor NaHS attenuated increased endothelial permeability and inflammation in murine lung specimens challenged by particulate matter inhalation [41]. In contrast, some reports demonstrated that decreased endogenous H_2_S production and altered sulfur metabolism reduced vascular permeability [42,43]. These currently available studies indicate that H_2_S signaling modulates EC barrier function in a context-dependent manner, and its potential disease-specific impact needs to be determined by targeted research [30]. To our knowledge, no such data exist about H_2_S in the context of SARS-CoV-2-mediated lung endothelial barrier disruption.

Recent findings have provided a rationale for considering the therapeutic implications of H_2_S donor molecules in COVID-19 therapy [44,45]. Our group and others have shown that H_2_S significantly attenuates the replication of several respiratory viruses and virus-induced inflammation [46,47]. Moreover, it has been speculated that H_2_S blocks SARS-CoV-2 entry into host cells by interfering with angiotensin-converting enzyme 2 (ACE2) and transmembrane protease serine 2 (TMPRSS2) expression [45]. In addition, impaired endogenous H_2_S availability is linked to cardiovascular [48], metabolic [49], and pulmonary diseases [50], which are all risk factors for developing severe COVID-19. Potential alterations in the endogenous H_2_S plasma level in COVID-19 patients are still under debate, but data suggest that impaired H_2_S availability contributes to COVID-19-associated endotheliopathy and a more severe outcome of this disease [51,52,53]. Furthermore, inhalation of the H_2_S donor, sodium thiosulfate, elicited protective effects in COVID-19 patients by reducing symptoms and accelerating recovery [54]. All these results suggest that H_2_S may exhibit beneficial effects in the pathomechanism of COVID-19, but the therapeutic potential of slow-releasing H_2_S compounds in lung endothelial barrier disruption associated with SARS-CoV-2 infection has never been explored.

In this study, we aimed to assess the effect of the slow-releasing H_2_S donor GYY4137 on the barrier function of a human lung microvascular EC monolayer in vitro, after challenging the cells with plasma samples from COVID-19 patients or inactivated SARS-CoV-2 virus. To our knowledge, these data are the first of their kind. We also evaluated inflammatory cytokine levels in the patients’ plasma and determined their correlation with disease severity and impact on the endothelial barrier function.

## 2. Materials and Methods

### 2.1. Human Plasma Samples

De-identified clinical plasma samples and clinical data of hospitalized COVID-19 patients were received from the University of Texas Medical Branch (UTMB) Biorepository for Severe Emerging Infections (BSEI). Samples were collected from consenting patients under the Clinical Characterization Protocol for Severe Emerging Infections (UNMC IRB # 146-20-FB/UTMB-IRB # 20-0066), PI, Dr. David Brett-Major, U. Nebraska Medical Center; UTMB site-PI, Dr. Susan McLellan. Note that multiple samples (longitudinal sampling) were collected from COVID-19 patients, classified as Mild (n = 7, total samples = 11), Moderate (n = 6, total samples = 12), Severe (n = 4, total samples = 12), or Critical (n = 9, total samples = 26) based on their oxygen therapy requirement by the following definitions: Mild—room air, Moderate—nasal cannula, Severe—non-invasive ventilation (continuous positive airway pressure/bilevel positive airway pressure/high-flow nasal cannula/large-reservoir oxygen mask), Critical—invasive ventilation (intubation; mechanical ventilation/extracorporeal membrane oxygenation). Patients typically moved through the various levels of oxygen devices and were classified into a disease severity category based on the highest level of oxygen delivery required. Control plasma samples (n = 20) from healthy subjects (one sample per subject) were collected under IRB protocol in Dr. Cardenas’ lab (HSC-GEN-09-0314). All samples were inactivated on dry ice by gamma irradiation (5 Mrad) prior to usage following institutionally approved Standard Operating Procedures for SARS-CoV-2 work.

### 2.2. Cell Culture

Human Lung Microvascular Endothelial Cells (HLMVECs, #540-05a), Attachment Factor Solution, and microvascular endothelial growth medium were purchased from Cell Applications (San Diego, CA, USA). HLMVECs were primary endothelial cells isolated from the lung capillaries of a 19-year-old Hispanic male. Cells were used up to passage six without losing their morphologic and phenotypic characteristics, per company recommendations. HLMVECs were grown in microvascular endothelial growth medium in a 5% CO_2_ atmosphere at 37 °C in coated cell culture flasks, dishes, and microplates following supplier’s instructions.

### 2.3. Virus

SARS-CoV-2 Omicron BA.1 (Lineage B.1.1.529 Strain: EHC_C19_2811C; GISAID: EPI_ISL_7171744) was provided by the World Reference Center for Emerging Viruses and Arboviruses (WRCEVA) at UTMB, propagated on TMPRSS2-expressing Vero E6 cells, sucrose purified and resuspended in HLMVEC basal medium with 1% BSA. The stock was then titrated by plaque assay as previously described [55]. Virus stock was then inactivated on dry ice by gamma irradiation (5 Mrad) before usage. Cultures from mock-infected cells were purified and processed similarly to vehicle control. All infectious work was performed in a biosafety level 3 laboratory (BSL3, Dr. Freiberg) at the Galveston National Laboratory, UTMB. The irradiated virus stock and corresponding vehicle control were transferred to a BSL-2 laboratory (Dr. Modis) for endothelial permeability assays. For this project, an irradiated equivalent dose of 5 × 10^4^ PFU was used per reaction in 200 µL volume (250 PFU/µL).

### 2.4. Reagents

Recombinant human TNF-α protein was purchased from R&D systems (Minneapolis, MN, USA). The slow-releasing H_2_S donor molecule, GYY4137, was purchased from Cayman (Ann Arbor, MI, USA). Bovine Serum Albumin (BSA) was purchased from Millipore Sigma (Burlington, MA, USA).

### 2.5. Bio-Plex Multiplex Immunoassay

Analysis of inflammatory markers in human plasma samples was performed using a Bio-Plex Pro Human cytokine group 1 panel 27-plex kit (Bio-Rad, Hercules, CA, USA) following the manufacturer’s instructions.

### 2.6. Endothelial Permeability Assay

An electrical impedance assay was adapted from the manufacturer’s protocol using the xCELLigence Real-Time Cell Analysis system (Agilent, Santa Clara, CA, USA). HLMVECs were seeded in a precoated 96-well cell culture plate equipped with gold electrodes (E-plate; Agilent, Santa Clara, CA, USA) at a density of 14,000 cells per well, predefined by previous experiments [56,57]. Cells were incubated under growth conditions for 48 h to form a confluent monolayer (indicated by a plateau of electrical impedance). The growth medium was then replaced by a prewarmed starvation medium containing 1% BSA and no growth supplements for 2 h before starting the treatment protocol. TNF-α and GYY4137 working solutions were prepared in starvation medium. For vehicle control of TNF-α and GYY4137 treatment, starvation medium was used. As controls for plasma treatment and virus challenge, growth medium and matching virus-free medium (respectively) were used. First, the HLMVEC monolayer was treated with human plasma, TNF-α, inactivated SARS-CoV-2 virus, or vehicle control. Thirty minutes later, GYY4137 or vehicle control was added to the wells, and the E-plates were further incubated in a 5% CO_2_ atmosphere at 37 °C for 24 h. All treatments were carried out in starvation medium, and each treatment group had 3–6 replicates on each plate. Plasma samples and inactivated virus stocks were used at 1:10 dilution (virus stock working concentration: 5 × 10^4^ PFU per each 200 µL reaction, 250 PFU/µL) with matching vehicle controls. TNF-α and GYY4137 were used at 10 ng/mL and 300 µM, respectively, unless otherwise indicated. Electrical impedance for each well was recorded as Cell Index (CI) every 10 min in real time throughout this process. CI readings were normalized (Normalized Cell Index, NCI) to the last recorded CI value before the first treatment to minimize well-to-well noise resulting from pretreatment differences (seeding, electrode sensitivity, etc.). During data analysis, NCI values were further normalized (Relative Normalized Cell Index, RNCI) to the negative control (treated with vehicle control only; control) readings from the same E-plate to account for plate-to-plate variability. Unless otherwise indicated, measurements at 12 h after treatment were analyzed and plotted.

### 2.7. Data and Statistical Analysis

Data are shown as mean ± SD. Statistical analyses included Rout outlier test (Q = 0.1%), D’Agostino–Pearson and Shapiro–Wilk normality tests, one-way, or two-way ANOVA followed by Tukey’s multiple comparisons test, Wilcoxon matched -pairs signed rank t-test, Kruskal–Wallis non-parametric one-way ANOVA test followed by Dunn’s multiple comparisons, and simple linear regression as indicated in figure legends. All statistical analysis was performed using GraphPad Prism 9 (version: Prism 9.5.1.) analysis software (GraphPad Software Inc., La Jolla, CA, USA). The experiments were repeated at least three times independently, with at least three replicates of each assay group or condition. A value of *p* ≤ 0.05 was considered statistically significant. In the Figures, “ns” indicates non-significant differences between two groups where needed for emphasis. Graphical abstract was created using BioRender.com.

## 3. Results

### 3.1. Patient Characteristics

In this study, we used de-identified clinical plasma samples and clinical data of hospitalized COVID-19 patients received from the UTMB Biorepository for Severe Emerging Infections. For the control, we used plasma from healthy subjects. COVID-19 patients were classified as Mild, Moderate, Severe, or Critical based on the highest level of their oxygen therapy requirement by the following definitions: Mild (n = 7)—room air, Moderate (n = 6)—nasal cannula (NC), Severe (n = 4)—non-invasive ventilation, and Critical (n = 9)—invasive ventilation. Demographic data, clinical characteristics, and initial laboratory findings for COVID-19 patients included in the study are summarized by disease severity groups in Table 1. Note that longitudinal data of clinical laboratory markers acquired during the patients’ hospitalization are not included in this table. Although the means of several clinical measurements show marked differences between patient groups at the time of admission, none of these differences reached statistical significance.

### 3.2. Plasma Cytokine/Chemokine Levels Show a Strong Correlation with Disease Severity

**Figure 1:**
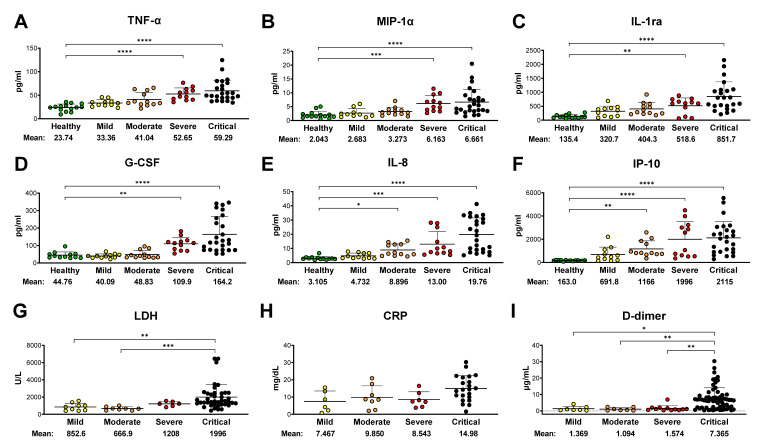
Disease severity positively correlates with plasma cytokine profiles and routine laboratory data. (**A**–**F**) Cytokine levels in plasma samples from COVID-19 patients and healthy volunteers were measured using Bio-Plex Pro Human Cytokine 27-plex Assay (Bio-Rad) and plotted grouped by disease severity. Only the statistical differences compared to the healthy control group are highlighted in these panels; (**G**–**I**) clinical laboratory marker measurements (all available longitudinal data; normal reference range: LDH < 280 U/L; CRP < 1 mg/dL; D-dimer < 0.5 µg/mL) of the same patient cohort were plotted grouped by disease severity. All statistical differences found between patient groups are labeled. All results in this figure are presented as dot plots of individual mean values for each sample. Bars represent group means and standard deviations. The statistical significance was assessed by Kruskal–Wallis non-parametric one-way ANOVA test followed by Dunn’s multiple comparisons. *, *p* < 0.05; **, *p* < 0.01; ***, *p* < 0.001; ****, *p* < 0.0001.

Elevated plasma levels of inflammatory markers (including tumor necrosis factor-alpha (TNF-α), macrophage inflammatory protein-1 alpha (MIP-1α), interleukin 8 (IL-8), interferon-gamma-induced protein 10 (IP-10), and others) have been reliably associated with more severe disease and serious symptoms including alveolar–capillary barrier disruption and intra-alveolar hemorrhage [58,59,60,61]. Based on these reports, we first sought to correlate disease severity with plasma biomolecules using the Bio-Plex Pro Human Cytokine 27-plex Assay (Bio-Rad). Interestingly, the plasma levels of ten signaling molecules (TNF-α, MIP-1α, interleukin-1 receptor antagonist (IL-1ra), granulocyte colony-stimulating factor (G-CSF), IL-8, IP-10, interferon-gamma (IFN-γ), monocyte chemoattractant protein-1 (MCP-1), platelet-derived growth factor (PDGF), and vascular endothelial growth factor (VEGF)) were found to be significantly elevated in patient samples compared to healthy controls, but only six (TNF-α, MIP-1α, IL-1ra, G-CSF, IL-8, and IP-10) showed a gradually rising trend of mean values in correlation with disease severity (Figure 1A–F). Remarkably, none of them were found to be significantly elevated in the mild plasma group compared to samples of healthy individuals, and four of them (TNF-α, MIP-1α, IL-1ra, and G-CSF) only showed a significant increase in severe and critical samples (Figure 1A–D). IL-8 and IP-10 demonstrated the most consistent correlation with disease severity, presenting significantly increased levels in moderate, severe, as well as critical samples (Figure 1E,F). Additionally, elevated expression levels of MCP-1, PDGF, VEGF, and IFN-γ exhibited only a partial correlation with disease severity.

### 3.3. Clinical Laboratory Markers Are Elevated in COVID-19 Patients’ Blood

Serum levels of lactate dehydrogenase (LDH), C-reactive protein (CRP), and D-dimer are routinely tested in hospitalized COVID-19 patients to assess acute inflammation, blood clotting tendencies, and increased tissue damage, respectively. We plotted all available longitudinal records of these clinical markers for each patient group (Figure 1G–I) to evaluate their correlation with disease severity and to compare them to the cytokine/chemokine profiles established earlier. Note that these measurements for the healthy control group were not available; however, comparing these results to the normal reference range (LDH < 280 U/L; CRP < 1 mg/dL; D-dimer < 0.5 µg/mL) [62,63,64] revealed that all three markers in all disease groups were elevated. In addition, LDH levels in the critical group were significantly increased even compared to the mild and moderate groups (Figure 1G). None of the groups presented distinct and significantly different CRP readings, although the critical group had a markedly higher mean value than the others. Elevated D-dimer levels correlated better with the critical categorization as values for that group were significantly higher relative to the severe, moderate, and mild groups. Overall, the three clinical markers together clearly distinguished the critical patient group from the others, but the close correlation of gradually increasing levels with disease severity found with inflammatory cytokines and chemokines was not observed in these tests.

### 3.4. GYY4137 Treatment Improves Endothelial Barrier Function

**Figure 2:**
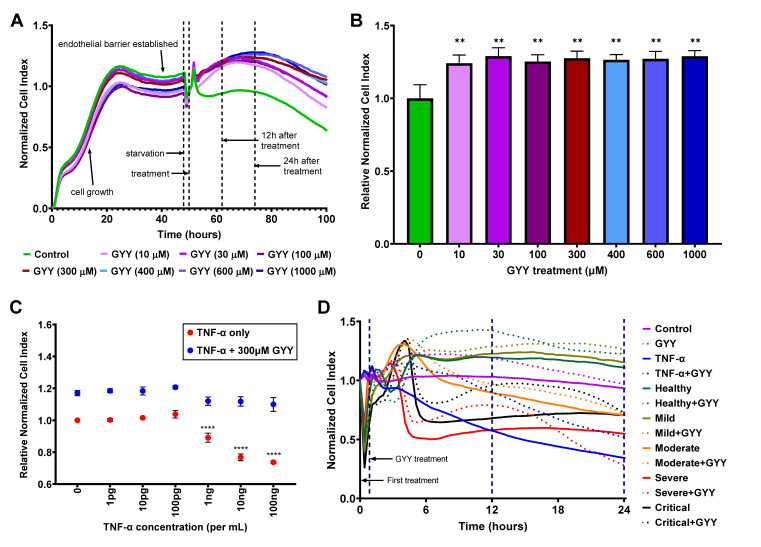
Exogenous H_2_S released by GYY4137 increases endothelial barrier function. HLMVECs were seeded on E-plates and incubated in growth medium for 48 h to form a confluent monolayer, then starved for 2 h before treatment. The effects of biomolecules and patient plasma treatment on the barrier function were monitored by electrical impedance measurements (Cell Index) using the xCELLigence Real-Time Cell Analysis system. Higher Cell Index, as well as Normalized and Relative Normalized Cell Index (described in Materials and Methods) values, represent increased barrier function. (**A**,**B**) GYY4137 treatment alone raises barrier function in all tested concentrations. Representative ribbon plots (mean of 3–6 replicates of each condition) of different GYY4137 concentrations (**A**) and histogram (mean ± SD, n = 3–9) of normalized data 12 h after treatment (**B**) are presented. Statistical differences for each GYY4137 concentration compared to control at 12 h are labeled. (**C**) GYY4137 treatment attenuates TNF-α–induced endothelial barrier disruption. Data are shown as mean ± SD of 3–6 measurements 12 h after treatment. Point 0 (zero control) red dot indicates no treatment, blue dot indicates GYY4137 treatment alone. Statistical differences compared to corresponding zero control are labeled. (**D**) GYY4137 enhances endothelial barrier function at 12 h after treatment with plasma samples. Ribbon plots show all measurements (as mean of 3–6 replicates of a single sample of each group) for 24 h after the first treatment. Thirty-minute, 12 h, and 24 h time points are marked by blue, dashed vertical lines. The statistical significance was assessed by (**B**) non-parametric Mann–Whitney U tests or (**C**) two-way ANOVA, followed by Tukey’s multiple comparisons test. **, *p* < 0.01; ****, *p* < 0.0001; GYY, GYY4137.

In our next set of experiments, we assessed the effects of biomolecules and patient plasma treatment on the barrier function of a confluent HLMVEC monolayer modeling the lung endothelial barrier in vitro, using the xCELLigence Real-Time Cell Analysis system (Agilent). This method uses electrochemical impedance measurements (Cell Index, CI) to determine the permeability of the endothelial monolayer with CI values, as well as Normalized and Relative Normalized Cell Index values (NCI and RNCI, respectively, as described in the Section 2). Higher CI, NCI, and RNCI values represent increased barrier function and a more intact endothelial monolayer. Reduced CI values show a compromised endothelial barrier and increased endothelial permeability. Since this study aimed to evaluate the potential beneficial effects of exogenous H_2_S on the lung microvascular endothelial barrier in COVID-19 patients, first, we tested the impact of H_2_S alone on our model. Using different concentrations (10–1000 µM) of the slow-releasing H_2_S donor, GYY4137, we established the real-time NCI curves of these treatments (Figure 2A) and determined their effects on the endothelial barrier at the 12 h time point after treatment (Figure 2B). We found that the real-time NCI curves for all tested GYY4137 concentrations consistently ran above the mock-treated control curve throughout the monitored 50 h long period after treatment (Figure 2A). Considering that under the starvation conditions of the assay the NCI of the untreated control (Figure 2A,B; green line and bar, respectively) started to fall 24 h after treatment, we limited the use of the assay to 24 h in all following experiments. We also determined that treatment with all tested GYY4137 concentrations significantly raised the RNCI compared to the control (Figure 2B) without any changes in cell viability and cytotoxicity. Based on these results and earlier studies [65], we selected a 300 µM concentration of GYY4137 to test its barrier-protective effect in combination with treatments with other agents. To verify the functionality and biological relevance of our model, we next attempted to mimic the loss of endothelial barrier integrity using TNF-α as the control since it has repeatedly been shown to interfere with the ECs’ tightness and cause increased barrier permeability [66,67,68]. We treated the HLMVEC monolayer with increasing concentrations of TNF-α (1 pg/mL–100 ng/mL) followed by 300 µM GYY4137 or mock treatment 30 min later. We found that 1–100 pg/mL TNF-α did not alter the RNCI, while 1–100 ng/mL TNF-α treatment resulted in decreased RNCI values in a dose-dependent manner at 12 h (Figure 2C) without increasing cellular toxicity. We measured very similar RNCI values after 24 h as well. Importantly, treating the cells with 300 µM GYY4137 30 min after TNF-α treatment completely restored RNCI levels suggesting the reestablishment of intercellular junctions. Altogether, these data verified that our human lung endothelial barrier model using the xCELLigence system is adequate to study changes in endothelial barrier permeability when subjected to various challenges.

### 3.5. Human Plasma Treatment Alters Endothelial Barrier Function, and GYY4137 Modifies These Effects

After establishing the impact of GYY4137 and TNF-α treatments, we evaluated the effects of patient plasma treatment on the HLMVEC monolayer. Plasma treatments resulted in significant alterations in NCI levels (Figure 2D), and each patient plasma sample produced a unique and robust EC barrier pattern recuperated very closely by replications (n = 3–6). Based on our previous results, we used 10 ng/mL TNF-α treatment as a positive control. We observed that some of the plasma treatments resulted in a comparable level of endothelial barrier disruption to TNF-α (continuous blue line), especially up to 12 h after treatment (continuous red and black lines corresponding to a severe and a critical sample, respectively). Interestingly, 300 µM GYY4137 treatment (dotted lines of the corresponding color) significantly alleviated the endothelial barrier disruption caused by plasma treatments at 12 h, and most of this beneficial effect was diminished at 24 h. None of the treatments caused detectable cytotoxicity measured by LDH-assay. Most importantly, adding GYY4137 after treating the HLMVECs with human plasma significantly increased EC barrier function, suggesting the barrier-protective impact of H_2_S donation.

### 3.6. Endothelial Barrier Disruption Caused by Plasma from COVID-19 Patients Does Not Correlate with Disease Severity or Plasma Cytokine/Chemokine Levels

**Figure 3:**
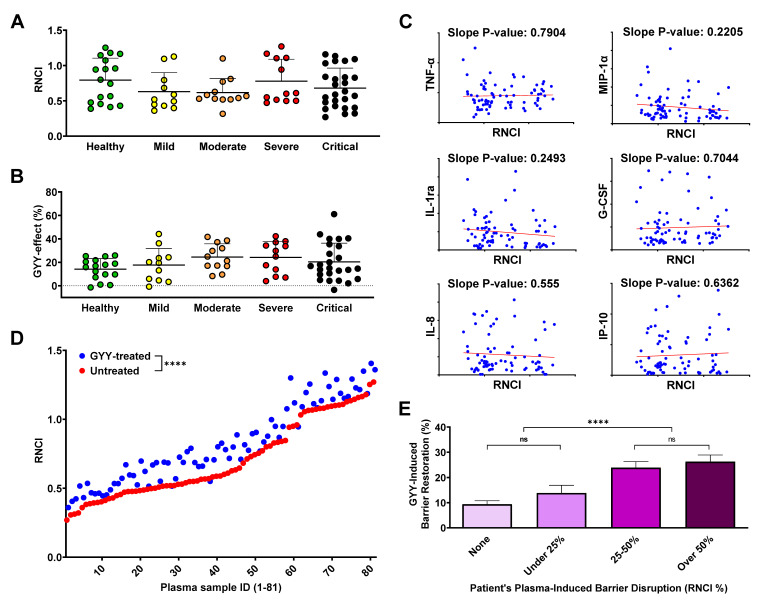
GYY4137 treatment ameliorates endothelial barrier disruption regardless of disease status. (**A**,**B**) Barrier function of an HLMVEC monolayer after treatment with COVID-19 or healthy plasma samples and a second treatment using 300 µM GYY4137 was monitored by electrical impedance measurements using the xCELLigence Real-Time Cell Analysis system. Results are expressed as Relative Normalized Cell Index (RNCI, described in Materials and Methods). Data were collected from at least three independent experiments for each sample (n = 3–6). RNCI values of HLMVEC monolayer after 12 h incubation with healthy or COVID-19 plasma samples (**A**) and the effect of GYY4137 on the RNCI of the plasma-treated HLMVEC monolayer at 12 h (**B**) were plotted by disease severity. Data are presented as dot plots of mean values from 3–6 repeated measurements of each sample. Bars represent group means and standard deviations. No statistical differences among disease severity groups were found. (**C**) Cytokine levels in the plasma samples used for barrier function experiments were measured using Bio-Plex Pro Human Cytokine 27-plex Assay (Bio-Rad) and plotted against the corresponding RNCI values at 12 h in scatterplots. Slope *p*-values were calculated to determine statistically significant levels of correlation. None were found. (**D**) Treatment efficacy plot showing RNCI of HLMVEC monolayer after plasma treatment with or without the addition of GYY4137. Dots represent means of 3–6 measurements for each treatment obtained at the 12 h timepoint. Statistical difference between RNCI values of untreated and GYY-treated samples is shown. (**E**) The effect of GYY4137 treatment for each plasma sample was plotted grouped by the level of plasma-induced barrier disruption. Bars represent group means + SD of all measurements (3–6 measurements per sample) 12 h after treatment. Selected statistical differences among groups are labeled. The statistical significance was assessed by Kruskal–Wallis non-parametric one-way ANOVA test followed by Dunn’s multiple comparisons (**A**,**B**), simple linear regression (**C**), 2-tailed, non-parametric, Wilcoxon matched-pairs signed rank t-test (**D**), and one-way ANOVA followed by Tukey’s multiple comparisons test (**E**). ****, *p* < 0.0001; ns, *p* > 0.05; GYY, GYY4137.

The recent literature demonstrated various levels of correlation between endothelial barrier disruption and the plasma cytokine levels and/or disease severity of patients with different pathological conditions, including COVID-19 [59,69,70]. Surprisingly, when we compared the effect of plasma from different disease severity groups on the RNCI of the HLMVEC monolayer, we found no statistical differences at 12 (Figure 3A) or 24 h, suggesting that our model was equally responding to samples from healthy individuals and COVID-19 patients. Similarly, when we calculated the barrier-altering effect of GYY4137 treatment after plasma treatment, we only detected a slightly increasing trend towards the more severe disease groups, but this trend did not reach the level of statistical difference (Figure 3B). Moreover, none of the biomolecules that showed a strong correlation with disease severity in experiments described earlier (Figure 1A–F) presented correlation with the RNCI after plasma treatment (Figure 3C). In fact, when we matched the barrier-disrupting effect of each plasma sample with its cytokine/chemokine profile, none of the inflammatory signaling molecules assessed in this study correlated with the corresponding RNCI values. Altogether, our results suggest that the change in endothelial barrier permeability in our model is neither linked to COVID-19 disease severity nor the individual plasma concentration of any of the inflammatory markers analyzed.

### 3.7. GYY4137 Increases Endothelial Barrier Function in a Disruption-Dependent Manner

Next, we evaluated the effect of GYY4137 by plotting the RNCI values of plasma- and GYY4137-treated samples against the corresponding RNCI results of samples treated with plasma only. We found a significant overall RNCI increase at 12 h as a result of GYY4137 treatment (Figure 3D) and, to a much lesser extent, at 24 h as well. Importantly, this analysis demonstrated that adding GYY4137 after treating the HLMVECs with human plasma significantly elevated EC barrier function, suggesting that GYY4137 combated plasma-induced barrier disruption. We then further explored the potential factors upon which the effect of GYY4137 treatment may depend. We found that greater initial barrier damage was met with a relatively greater healing effect by the administration of GYY4137 (Figure 3E). As a result, when we grouped our plasma samples based on their initial barrier-damaging effect (regardless of which disease severity group the patient belonged to), the more damaged groups of samples benefited more from GYY4137 treatment. Interestingly, instead of a gradual increase in restoring endothelial barrier integrity, we observed a relatively sharp increase at approximately 25% barrier disruption and a milder gradual rising trend both below and after this threshold.

### 3.8. Inactivated SARS-CoV-2 Omicron BA.1 Increases Endothelial Barrier Permeability, Which Can Be Prevented by GYY4137 Treatment

**Figure 4:**
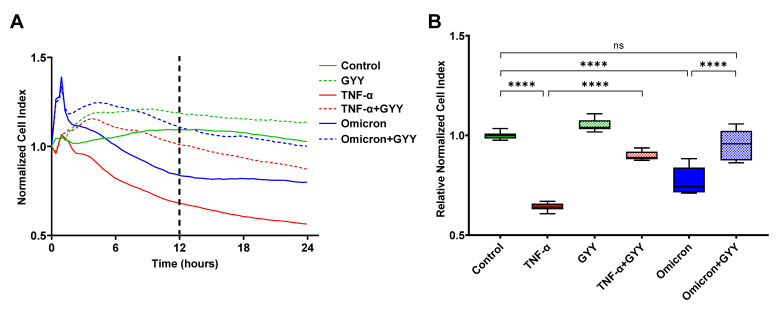
GYY4137 treatment restores endothelial barrier integrity disrupted by inactivated SARS-CoV-2 Omicron BA.1. Barrier function of an HLMVEC monolayer after treatment with inactivated SARS-CoV-2 or 10 ng/mL TNF-α and a second treatment with 300 µM GYY4137 was monitored by electrical impedance measurements using the xCELLigence Real-Time Cell Analysis system and expressed as Normalized Cell Index and Relative Normalized Cell Index (NCI and RNCI, respectively, described in Materials and Methods). Representative plots of three independent experiments are shown. (**A**) Ribbon plots show all NCI measurements (as mean of 6–12 replicates) for 24 h after the first treatment. The 12 h time point is marked by dashed vertical line. (**B**) Box and whiskers plots represent RNCI data 12 h after treatment (6–12 replicates per treatment). The box extends from the 25th to 75th percentiles of each sample set, the whiskers go down to the smallest value and up to the largest. The line in the middle of the box is plotted at the median. Selected statistical differences among treatments are labeled. The statistical significance was assessed by one-way ANOVA followed by Tukey’s multiple comparisons test using Graph Pad Prism 9. ****, *p* < 0.0001; ns, *p* > 0.05; GYY, GYY4137; Omicron, SARS-CoV-2 Omicron B.1.1.529.

A direct contribution of the SARS-CoV-2 virus to the endothelial barrier disruption observed in the lungs of COVID-19 patients has been debated since the beginning of the pandemic. Most recent studies have suggested that contact with viral proteins (most notably the spike protein and its S1 subunit) rather than viral infection of the endothelial cells may play a role in endothelial activation and barrier disruption [19,20]. Based on these studies, we assessed the impact of inactivated SARS-CoV-2 Omicron BA.1 (Omicron) in our in vitro model. The endothelial monolayer was treated with an equivalent of 5 × 10^4^ PFU per well or a similarly prepared mock (control). We found that compared to the vehicle control, virus treatment caused a significant decrease in NCI and RNCI values at both 12 and 24 h after treatment (Figure 4A,B). Specifically, the NCI curve of virus treatment ran parallel to the 10 ng/mL TNF-α control curve up to the 12 h time point causing approximately 60% as much decrease in barrier function (Figure 4A). Throughout the next 12 h, the virus-treated curve remained steady. Overall, inactivated virus treatment caused an approximately 20–25% increase in endothelial barrier permeability both at 12 and 24 h compared to the control suggesting a direct contribution of SARS-CoV-2 Omicron BA.1 proteins to lung endothelium damage. Similar to our results with plasma from COVID-19 patients, treating our lung endothelial barrier model initially damaged by inactivated virus challenge with GYY4137 (300 µM) significantly improved endothelial barrier function (Figure 4A,B). However, unlike with the plasma samples, this restorative effect by the H_2_S-donor did not diminish over time and remained at virtually identical levels to the mock-treated control up until the 24 h time point (Figure 4A). None of the treatments caused detectable cytotoxicity measured by LDH-assay. Most importantly, GYY4137 treatment prevented the disruption of endothelial barrier integrity caused by virus challenge (Figure 4B), altogether suggesting that GYY4137 exerts a therapeutic effect in lung endotheliopathy seen in COVID-19 patients.

## 4. Discussion

One of the novel findings of the present study is that treatment with GYY4137, a well-characterized, slow-releasing H_2_S donor, ameliorates endothelial barrier disruption caused by plasma samples from COVID-19 patients in vitro, regardless of disease severity. In our real-time in vitro assay model, patient plasma altered endothelial barrier permeability in a highly sample-specific manner, causing barrier damage comparable to TNF-α control, a cytokine known to disrupt endothelial junctions [66,67,68]. Surprisingly, plasma-induced barrier disruption did not correlate with disease status based on the patient’s oxygen requirement: Plasma from some patients with mild disease caused as much or even more damage as some critical patients’ plasma, while plasma from some other individuals in each disease severity group did not elicit any increase in endothelial barrier permeability or even trigger a decrease. Additionally, we determined the cytokine/chemokine profile of each plasma sample and found a high correlation between disease severity and the concentration of several biomolecules, most notably IL-8 and IP-10. In fact, their plasma levels mirrored increasing disease severity much closer than any of the three regularly used clinical markers, LDH, CRP, and D-dimer [71,72,73]. On the other hand, none of the cytokines and chemokines we assessed demonstrated correlation between plasma levels and the corresponding in vitro barrier function assay results. Finally, inactivated SARS-CoV-2 Omicron BA.1 virus particles elicited a very robust endothelial barrier disruption in our assay that was completely reversed by adding GYY4137. Taken together, these data characterize the effects of patient plasma and inactivated virus particles on the lung microvascular endothelial barrier and provide the basis for further efforts to develop novel treatment modalities that specifically target H_2_S signaling in COVID-19 patients.

In the fight against COVID-19, vaccine development and distribution, together with other preventive measures, are used as the first line of defense with limited success around the world [74,75]. Serving as the second line, the efficacy of therapeutic approaches in reducing morbidity and mortality of the disease has been gradually increasing, but there is still a great need for novel effective and inexpensive drugs to fill in the gaps of currently available treatment modalities, especially outside the highest-income countries [76,77]. In light of recent discoveries about its anti-inflammatory, vasculoprotective, and antiviral effects, H_2_S has been proposed as a potential defense against COVID-19 [44,45]. In this study, we aimed to assess the effect of an H_2_S-donor, GYY4137, on the barrier function of lung endothelial cells, after challenging them with plasma samples from COVID-19 patients, including non-survivors or inactivated SARS-CoV-2 virus. In parallel, we also sought to demonstrate any correlation between inflammatory markers in patient plasma and disease severity.

A damaged endothelial barrier and increased microvascular permeability are hallmarks of severe COVID-19 pathology, greatly contributing to disease severity and mortality [6,8,11]. H_2_S has been shown to modulate vascular permeability in several reports, and its effects on the endothelial barrier function have been suggested to be potentially disease- and organ-specific [30,36,38]. In this regard, we established a lung microvasculature-specific assay and tested the effects of the H_2_S-donor, GYY4137, in the context of COVID-19-associated biological samples (patient’s plasma and inactivated virus particles). We found a significant increase in barrier function (termed CI, NCI, and RNCI as described in the Section 2) 12 h after GYY4137 treatment regardless of the existence and nature of pretreatments. Interestingly, this effect was maintained, diminished, or even reversed at later time points depending on the initial treatment. These results are in line with recent reports showing that H_2_S inhalation or H_2_S-donor treatment reduced pathologically increased vascular permeability in the brains of rats after cardiac arrest [37] or in the lungs of mice after particulate matter inhalation [41], respectively. Remarkably, larger initial barrier disruption by COVID-19 patients’ plasma evoked a relatively greater barrier function increase by GYY4137 treatment. Beyond the obvious base-effect (from a lower base, the same nominal increase constitutes a higher percent), we found an unexplained phenomenon that a 25% or larger RNCI decrease caused by plasma treatment was followed by a significantly greater increase evoked by GYY4137 than when the initial barrier damage was smaller. This could be caused by some of the tight or adherens junctions between ECs that are preferentially restored first or faster by GYY4137 and confer different levels of connecting strength between cells [78,79], but to fully understand the reason for this bi-phasic effect will require further investigations. Taken together, our data support the notion that an increased level of H_2_S in the lung microenvironment, either by increased endogenous production or by pharmaceutical intervention, may be beneficial in severe COVID-19.

Another question we addressed was how the cytokine/chemokine profile of patients’ plasma, the endothelial barrier disruption caused by this plasma, and disease severity correlate with each other. As expected, we found higher levels of inflammatory cytokines and chemokines in the plasma of COVID-19 patients with more severe disease, confirming previously reported data [15,59,61]. Moreover, six (TNF-α, MIP-1α, IL-1ra, G-CSF, IL-8, IP-10) of the assessed signaling molecules demonstrated gradually increasing plasma levels consistent with disease severity, much more so than routine clinical markers, LDH, CRP, and D-dimer [71,72,73]. In fact, we found that while these markers (especially LDH and D-dimer) clearly distinguished the critical group from the others, they did not separate the other groups. While there are obvious advantages to recognizing the critical phase in the course of COVID-19 using blood tests designed to detect these molecules, a panel of the six cytokines/chemokines listed above could provide a better resolution to monitor disease progression. Surprisingly, some biomolecules that have been reported to be potential markers for COVID-19 disease severity, including VEGF [80,81], MCP-1 [60], IFN-γ [82], PDGF [81], and others, either did not or only partially correlated with disease severity, maybe due to the relatively small number (10–12) of samples in some of the groups. Nevertheless, our data support previous findings that monitoring blood cytokine/chemokine levels, especially for IL-8 [58,61] and IP-10 [60,83], could be used as additional biomarkers to help identify and manage COVID-19 patients with different disease severities. On the other hand, we found no correlation between plasma cytokine/chemokine levels and endothelial barrier disruption in our in vitro assay. In light of this finding, it is not surprising that the measured in vitro barrier disruption does not correlate with disease severity either. This counterintuitive result is, in fact, in line with the recent literature [84] dissecting the factors in COVID-19 patients’ plasma potentially causing endothelial barrier disruption. Kovacs-Kasa et al. verified that the factor(s) in patients’ plasma disrupting microvascular integrity were heat-labile, but no single or set of cytokine(s) could account for enhanced vascular permeability. They also disproved the potential role of ACE2-binding and complement factors C3a and C5a in the phenomenon. Recent studies implicated several molecular mechanisms, including the altered expression and function of adhesion and junction proteins (ICAM-1 and 2, VCAM-1, E- and P-selectin, claudins, occludins, VE-cadherin, Connexin-43, and others) [78,79,85], and/or pathologically modified signaling by integrins, TGF-β, complement, the glycocalyx, mitochondria, and (most recently) microRNAs [12,19,86,87], to contribute to lung endothelial barrier disruption, but the causative agents in the plasma initiating these processes are still widely debated. Since the plasma levels of the cytokines/chemokines assessed in this study are several magnitudes lower than necessary to significantly lower CI values in our assay (e.g., for TNF-α, 1–100 pg vs. 1–100 ng, respectively), a direct effect from these molecules in vitro could not be expected. However, lung tissue levels of these mediators are estimated to be potentially over 1000-fold higher than in plasma during severe inflammation [88,89], reaching the necessary levels for endothelial activation and barrier disruption in vivo. It is also worth noting that disease severity groups were solely based on the patients’ oxygen requirements without consideration of any other clinical characteristics. A more complex classification system including several clinical markers and symptoms could yield different results. Therefore, further in vitro and in vivo studies will be needed to resolve this debate.

Circulating virus particles and/or the viral spike protein in the patient’s blood have also been implicated in inducing increased microvascular permeability. The potential role and significance of the spike protein (or other viral proteins) in endothelial barrier disruption are still highly controversial [20,69,78,86,90] and not always assessed when using these assays. For example, endothelial damage has been reported in the lung after using (1) the spike protein that induced degradation of junction proteins [78] as well as altered integrin and transforming growth factor beta signaling [19], (2) the nucleoprotein that induced EC activation via Toll-like receptor 2 and mitogen-activated protein kinase signal pathways [13], and (3) non-infectious pseudovirus expressing the spike protein that compromised mitochondria and impeded endothelial NO synthase activity [86]. To this end, we tested the effects of inactivated SARS-CoV-2 Omicron BA.1 virus in our in vitro assay and demonstrated that challenging the HLMVEC monolayer by a 5 × 10^4^ PFU/well infective dose equivalent inactivated virus results in a drop in CI values comparable to the effect of 10 ng/mL TNF-α. We chose to work with a B.1.1.529 variant as this lineage was circulating in the population at the time of experimentation. Most importantly, inactivated virus-induced endothelial barrier disruption was completely reversed by 300 µM GYY4137. We speculate that the barrier-disruptive potential of the virus particle and/or the spike protein could be lineage- and even sublineage-dependent, explaining the inconsistent data about endothelial barrier disruption available from similar studies using proteins derived from Wuhan or WA1/2020 isolates [69]. For example, it has been well-demonstrated that Omicron lineages feature increased ACE2-affinity and immune evasion capabilities due to several mutations, most of which alter the antigenicity of the spike protein and at the same time modify its structure and function [91,92,93]. As a result of their unique virological features in comparison to other SARS-CoV-2 strains, Omicron variants exhibit less efficient TMPRSS2 usage, less spike cleavage, lower fusogenicity, and an altered entry mechanism [94,95]. Similarly, the use of different endothelial cells could also be the source of inconsistent findings because of the different genetic backgrounds of the original donors [69]. Further studies addressing virus–host cell interactions with respect to the spectrum of genetic variations in both could be warranted to better assess the clinical relevance of this pathomechanism.

Our study has certain limitations. First, the number of plasma samples per group was somewhat uneven; there were more critical and healthy samples available than samples belonging to the other three disease severity groups. This weakness of the study design may have introduced a bias towards more significant differences between the two larger sample groups than the others, but we do not believe that it fundamentally altered any of our findings. A follow-up study with larger sample numbers could verify our data. Second, we focused on only one aspect of COVID-19-associated endotheliopathies, the alterations of barrier function using ECs only. While a more complex study could put the results in more context, our simplified monoculture-based approach had the advantage of providing clear answers to some of the basic questions: (1) Are there factors in patient plasma capable of altering endothelial barrier function alone? (2) What correlations exist among the cytokine/chemokine profile of plasma samples, the endothelial barrier disruption caused by them, and disease severity? (3) Can inactivated virus alone, as a surrogate of using viral proteins, cause endothelial barrier disruption? Most importantly: (4) Does treatment with an H_2_S-donor provide beneficial effects against SARS-CoV-2-associated lung microvascular barrier disruption? Third, we only tested one virus variant and primary lung ECs from one donor as proof of concept. As discussed above, these choices have introduced a genetic bias for the virus–host cell interactions, and we believe that our results justify more comprehensive follow-up studies. Finally, we only tested the effects of a one-time treatment with a single H_2_S donor molecule, GYY4137. Other H_2_S-releasing agents may have more sustained pharmacological effects as recently reviewed by Szabo and Papapetropoulos [96]. Further studies will be necessary to clarify the effects of repeated treatments using several different H_2_S-releasing compounds to verify whether potential clinical trials could be warranted for pharmacological increase/stabilization of the endothelial barrier as a third pillar for the treatment of COVID-19 in addition to immunomodulators and antivirals.

## 5. Conclusions

Overall, our data demonstrate that treatment with H_2_S-releasing compounds has the potential to ameliorate SARS-CoV-2-associated lung endothelial barrier disruption. Although much work remains to be conducted to fully understand and dissect the molecular mechanisms involved as well as the therapeutic implications of this approach in treating COVID-19, this work provides the basis for future investigations.

## 6. Patents

O.E. and A.N.F. reported a patent for treating viral infections using hydrogen sulfide donors (US-9504701-B2).

## Figures and Tables

**Table 1:** Demographic data, clinical characteristics, and laboratory findings of COVID-19 patients included in the study. Data sets of clinical measurements at the time of admission (BMI, temperature, oxygen saturation, respiration rate, LDH, CRP, D-dimer) were analyzed for statistical differences between disease severity groups. None were found. The statistical significance was assessed by Kruskal–Wallis non-parametric one-way ANOVA test followed by Dunn’s multiple comparisons and one-way ANOVA followed by Tukey’s multiple comparisons test. ^a^ Highest level of oxygen delivery required; BMI, body mass index; LDH, lactate dehydrogenase; CRP, C-reactive protein.

Disease Severity	Mild (n = 7)	Moderate (n = 6)	Severe (n = 4)	Critical (n = 9)
Room air ^a^ (%)	100.0%	0.0%	0.0%	0.0%
Nasal cannula ^a^ (%)	0.0%	100.0%	0.0%	0.0%
Non-invasive ventilation ^a^ (%)	0.0%	0.0%	100.0%	0.0%
Invasive ventilation ^a^ (%)	0.0%	0.0%	0.0%	100.0%
Male (%)	57.1%	33.3%	75.0%	66.7%
Age at admission, median (years)	55	59	54	55
Hispanic ethnicity (%)	28.6%	33.3%	0.0%	33.3%
White race (%)	57.1%	83.3%	75.0%	55.6%
Black race (%)	42.9%	16.7%	25.0%	44.4%
Days admitted, median (days)	5	7	21	36
On dexamethasone (%)	14.3%	50.0%	75.0%	100.0%
Taking remdesivir (%)	57.1%	83.3%	100.0%	100.0%
No antiviral used (%)	42.9%	16.7%	0.0%	0.0%
COVID-vaccinated (%)	0.0%	0.0%	0.0%	0.0%
Discharged alive (%)	100.0%	100.0%	75.0%	11.1%
Transferred to another facility (%)	0.0%	0.0%	25.0%	33.3%
Death (%)	0.0%	0.0%	0.0%	55.6%
**Clinical characteristics at admission (mean ± SD)**				
BMI	34.80 ± 6.41	31.34 ± 4.50	39.53 ± 15.30	37.8 ± 9.24
Body weight (kg)	99.07 ± 19.04	85.98 ± 9.45	112.71 ± 22.38	113.42 ± 32.73
Temperature (degrees, °C)	37.10 ± 0.43	37.46 ± 1.13	38.00 ± 0.69	37.35 ± 1.00
Oxygen saturation (%)	96.14 ± 2.96	95.16 ± 2.22	90.75 ± 14.08	89.33 ± 4.35
Respiration rate (breaths/minute)	21.85 ± 5.04	21.50 ± 3.56	27.75 ± 17.63	26.77 ± 8.65
LDH (U/L)	750.45 ± 466.14	785.25 ± 204.77	1167.33 ± 322.76	1065.5 ± 389.10
CRP (mg/dL)	8.50 ± 6.14	13.67 ± 6.59	10.36 ± 5.31	14.49 ± 5.77
D-dimer (µg/mL)	1.31 ± 1.48	0.57 ± 0.52	0.78 ± 0.36	1.63 ± 1.77

## Data Availability

Raw data generated in the current project are available, upon request, from the author of the correspondence (K.M.).
